# Comparative Transcriptome Analysis Identified Candidate Genes for Late Leaf Spot Resistance and Cause of Defoliation in Groundnut

**DOI:** 10.3390/ijms22094491

**Published:** 2021-04-26

**Authors:** Sunil S. Gangurde, Spurthi N. Nayak, Pushpesh Joshi, Shilp Purohit, Hari K. Sudini, Annapurna Chitikineni, Yanbin Hong, Baozhu Guo, Xiaoping Chen, Manish K. Pandey, Rajeev K. Varshney

**Affiliations:** 1 Center of Excellence in Genomics & Systems Biology (CEGSB), International Crops Research Institute for the Semi-Arid Tropics (ICRISAT), Hyderabad 502324, India; g.sunil@cgiar.org (S.S.G.); j.pushpesh@cgiar.org (P.J.); purohit.shilp@gmail.com (S.P.); h.sudini@cgiar.org (H.K.S.); a.chitikineni@cgiar.org (A.C.); 2 Department of Genetics, Osmania University, Hyderabad 500007, India; 3 Department of Biotechnology, University of Agricultural Sciences, Dharwad 580005, India; nayaksn@uasd.in; 4 Crops Research Institute, Guangdong Academy of Agricultural Sciences, Guangzhou 510640, China; hongyanbin@gdaas.cn (Y.H.); chenxiaoping@gdaas.cn (X.C.); 5 USDA-ARS, Crop Genetics and Breeding Research Unit, Tifton, GA 31793, USA; baozhu.guo@usda.gov

**Keywords:** late leaf spot, *Arachis hypogaea*, *Nothopassalora personata*, RNA-seq, differentially expressed genes, pathway analysis, defoliation, peanut

## Abstract

Late leaf spot (LLS) caused by fungus *Nothopassalora personata* in groundnut is responsible for up to 50% yield loss. To dissect the complex nature of LLS resistance, comparative transcriptome analysis was performed using resistant (GPBD 4), susceptible (TAG 24) and a resistant introgression line (ICGV 13208) and identified a total of 12,164 and 9954 DEGs (differentially expressed genes) respectively in A- and B-subgenomes of tetraploid groundnut. There were 135 and 136 unique pathways triggered in A- and B-subgenomes, respectively, upon *N. personata* infection. Highly upregulated putative disease resistance genes, an RPP-13 like (*Aradu.P20JR*) and a NBS-LRR (*Aradu.Z87JB*) were identified on chromosome A02 and A03, respectively, for LLS resistance. Mildew resistance Locus (MLOs)-like proteins, heavy metal transport proteins, and ubiquitin protein ligase showed trend of upregulation in susceptible genotypes, while tetratricopeptide repeats (TPR), pentatricopeptide repeat (PPR), chitinases, glutathione S-transferases, purple acid phosphatases showed upregulation in resistant genotypes. However, the highly expressed ethylene responsive factor (ERF) and ethylene responsive nuclear protein (ERF2), and early responsive dehydration gene (ERD) might be related to the possible causes of defoliation in susceptible genotypes. The identified disease resistance genes can be deployed in genomics-assisted breeding for development of LLS resistant cultivars to reduce the yield loss in groundnut.

## 1. Introduction

Groundnut or peanut (*Arachis hypogaea* L.) is a self-pollinated tetraploid oilseed legume crop and is cultivated on 34.1 million hectares (Mha) with an annual production of 66.3 million tons with productivity of 2.17 tons/ha during 2019 [1]. Groundnut with 20 chromosomes (2n = 4x = 40, AABB) and genome size of 2.6 Gb [2,3,4] is one of the important food crops of semi-arid tropics because of high quality edible oil, protein, minerals and vitamins like biotin, niacin, folate and vitamin E [5]. Global productivity of this crop is reduced substantially due to the occurrence of the foliar disease late leaf spot (LLS) caused by *Nothopassalora personata* (Berk. and M. A. Curtis) S. A. Khan and M. Kamal (syn. *Cercosporidium personatum* (Berk. and M. A. Curtis) Deighton) [6,7,8,9]. It is estimated that LLS alone can cause 50–70% yield penalty if the crop is not protected by applying fungicides [10]. Defoliation is a common symptom that is observed in LLS infected leaves which adversely affects the haulm quality and fodder as well as pod yield [11,12]. Although the LLS infection in fields can be managed by multiple application of fungicide in the growing season [13], to some extent, however, it adds extra cost of cultivation in addition to environmental pollution and affects soil microflora ecosystem. For instance, 0.15% Tebuconazole is a best fungicide to reduce the LLS disease intensity and can save up to 67% pod yield [13]. However, the application of Tebuconazole reduces the activities of enzymes in the soil, soil biomass and affects the structure of soil ecosystem [14,15].

Screening of cultivated gene pool identified several partial resistant sources for LLS resistance [16,17] which encouraged researchers to look for stable resistance source among interspecific lines derived from known wild resistance sources [18]. An interspecific LLS resistant genotype, ICGV 86855 (developed from cross between *A. hypogaea* and resistant *A. cardenasii*) was used to breed GPBD 4, a foliar disease resistant (FDR) variety [16] which later on become the resistant check in all the national trials for FDR in India. Development and use of a recombinant inbred line (RIL) population using GPBD 4 as resistant parent facilitated genetic mapping and identification of quantitative trait loci (QTLs) [19,20] and candidate genes [4,21] as well as diagnostic markers which are being currently used for performing marker-based early generation selection (MEGS) to improve LLS resistance [7].

Availability of reference genomes allowed sequencing-based trait mapping such as QTL-seq approach using RIL population (TAG 24 × GPBD 4) leading to identification of 25 candidate genes for LLS resistance in a genomic region of 3.06 Mb (131 to 135 Mbp) on chromosome A03 of diploid progenitor genome [7] which was later detected on chromosome chr13 (B03) upon using tetraploid reference genome [4]. The same RIL population was also subjected to ddRADSeq which too confirmed QTLs for LLS and rust resistance on 1.4- and 2.7-Mb genomic regions on chromosomes A02 and A03, respectively [22]. Another study using a different source performed whole genome sequencing of complete RIL population (Tifrunner × GT-C20) and identified major QTLs on chromosome A05 and B03 with phenotypic variance ~47% in US germplasm [23]. These genetic mapping studies confirmed the genomic region on chromosome A03/B03 responsible for LLS resistance and the linked SSR/SNP markers [7] were used in marker assisted selection to develop LLS resistance varieties [9,24,25,26].

Despite having information on genomic regions and linked markers, there is no information on the candidate resistance genes with differential gene expression analysis. Transcriptome analysis also gives an advantage to study the expression of genes in previously reported QTL region [27]. Recently, a comparative transcriptome analysis between JL24 (susceptible) and GPBD 4 (resistance) was performed to identify the differentially expressed genes (DEGs) for rust resistance under infection of *Puccinia arachidis* [28] and early leaf spot resistance under infection of *Cercospora arachidicola* [29]. An introgression line, ICGV 13208, used in the present study was developed by introgressing the resistance genomic region from GPBD 4 in TAG 24 [30]. In this study, we performed a comparative transcriptome analysis for LLS resistance between introgression line (ICGV 13208) and its parents TAG 24 (susceptible) and GPBD 4 (resistance) to discover the genome-wide differentially expressed genes. Studying the comparative transcriptome of introgression lines with parental genotypes provided evidence regarding the expression of candidate genes that are present in the associated genomic regions. The information available on potential candidate genes identified through this study may be advantageous for development of functional markers and genome editing for improving LLS resistance in groundnut.

## 2. Results

### 2.1. RNA Sequencing and Development of Transcriptome Assembly

A resistant genotype (GPBD 4), a susceptible genotype (TAG 24) and an introgression line (ICGV 13208) carrying LLS resistant QTL, derived from the marker-assisted backcrossing between TAG 24 (recurrent parent) × GPBD 4 (donor parent) were used for transcriptome analysis under control (non-inoculated) and inoculated condition (inoculated with *N. personata*). The conditions included one stage before and seven stages post inoculation (0, 1DPI, 2DPI, 3DPI, 7DPI, 21DPI, 35DPI, 50DPI). In this way, 48 samples representing (3 genotypes × 2 treatments (stressed and control) × 8 stages) were used for comparative transcriptome analysis. Pair end sequencing [2 × 100 bp] of these 48 samples generated a total of 1459.5 million pair reads. After rigorous filters, such as the reads with adapter sequences, short reads and reads with too many ambiguous (N) bases during quality analysis, 1308.6 million paired reads (around 90% of total reads) were retained for global gene expression profile and differential gene expression studies. On an average, 78.2% reads were mapped on both subgenomes but the highest percentage (79.8%, i.e., 1044.8 million reads) of mapping was achieved for B-subgenome (Table S1). The sequencing data generated in present study have been deposited in National Center for Biotechnology Information Sequence Read Archive (NCBI-SRA) database with the Bio Project ID-PRJNA660596.

**Figure 1:**
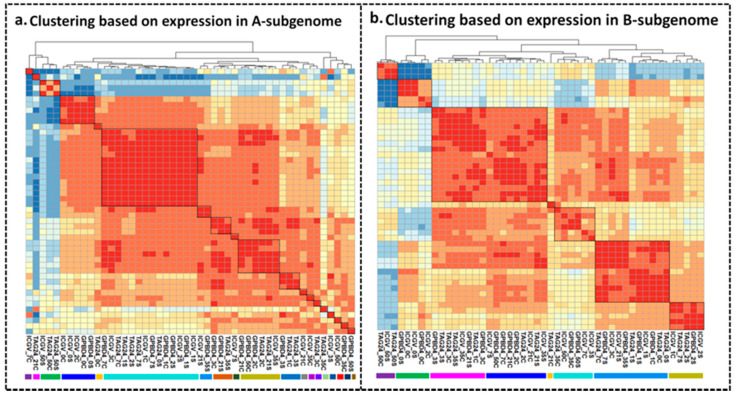
Pairwise correlation between stressed and control tissues using global gene expression patterns in A- and B-subgenomes. Genes with a normalized expression level FPKM > 1 in at least one of the 48 tissues analyzed were log2. +1 transformed before analysis and were designated as expressed. (**a**) Clustering based on expression in A-subgenome. (**b**) Clustering based on expression in B-subgenome.

The samples were clustered using the expression values of all DEGs from both subgenomes individually. On the basis of expression values of DEGs in A-subgenome, majority of samples under stress at 1DPI, 2DPI, 7DPI were found clustered together across all genotypes. Control and stressed samples at 0DPI of GPBD 4 and ICGV 13208 were found clustered together along with ICGV 13208 control at 2DPI. Control and stressed samples of TAG 24 at 50DPI grouped together with ICGV 13208 stressed at 50DPI. Stressed samples of GPBD 4 and ICGV 13208 at 21DPI and 35DPI also grouped together. Several of the samples did not show any grouping due to very unique transcriptome abundance as compared to other samples (Figure 1a).

Clustering with expression values in B-subgenome showed majority of the samples clustered into four major groups. Control samples at stages 2DPI, 7DPI, 21DPI of GPBD 4 and TAG 24 clustered together. At 3DPI, control as well as stressed samples of TAG 24 and GPBD 4 grouped together. This indicated that at 3 DPI, there may not be significant changes at transcriptome level in B-subgenome of susceptible and resistant genotypes. All control samples at 21DPI, 35DPI, 50DPI of TAG 24 and ICGV 13208 were clustered together with GPBD 4 stressed at 50DPI indicated that GPBD 4 has strong resistance against *N. personata* even at 50DPI. All stressed samples at 2DPI of GPBD 4, ICGV 13208 and TAG 24 were clustered together. Control samples of ICGV 13208 and GPBD 4 at 0DPI were clustered together indicating similar expression pattern in GPBD 4 and in ICGV 13208. All stressed samples at 1DPI of TAG 24, GPBD 4 and ICGV 13208 clustered together with stressed and control samples at 0DPI of TAG 24 and control at 1DPI of GPBD 4 (Figure 1b).

### 2.2. Genome-Wide Differential Gene Expression Patterns in Resistant and Susceptible Genotypes

**Figure 2:**
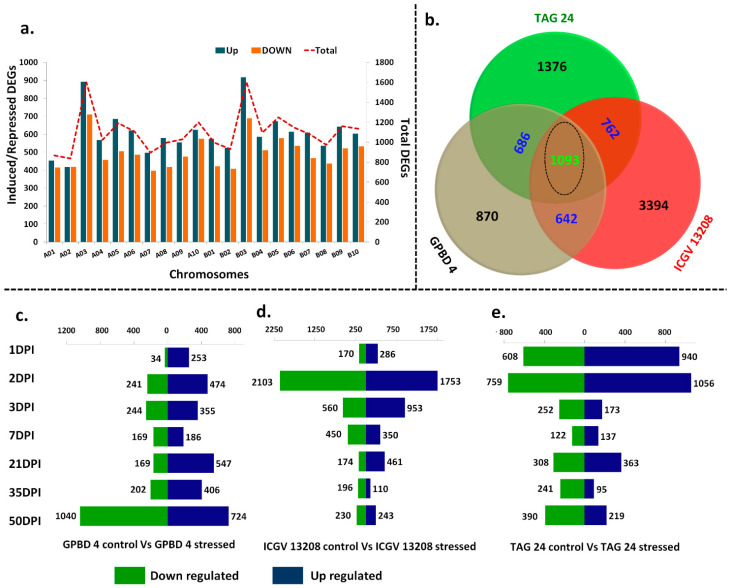
Summary of globally differentially expressed genes and DEGs between control and stressed samples of same genotypes at different stages. (**a**) Genome wide differentially expressed genes including upregulated (blue) and downregulated (orange) plotted on primary axis. The upper dotted line indicates total DEGs (up- and downregulated) plotted on secondary axis. (**b**) Venn diagram representing the globally expressed common, unique and core set of DEGs between GPBD 4, TAG 24 and ICGV 13208. The day wise up- and downregulated DEGs are presented in (**c**) GPBD 4 control vs. GPBD 4 stressed, (**d**) ICGV 13208 control vs. ICGV 13208 stressed, (**e**) TAG 24 control vs. TAG 24 stressed.

The genes with least transcript abundance (<1 FPKM) in all the samples were filtered out and not used for further analysis. With this criterion, a total of 22,670 genes in A-subgenome and 24,349 genes in B-subgenome were found to be expressed with FPKM value ≥ 1. The fold-change (log2 fold) of each gene was calculated across combinations of resistant and susceptible genotypes at disease development and symptom development stages and the gene was said to be differentially expressed when the log2 fold-change value was ≥2 (induced) or ≥−2 (repressed). With these criteria, a total of 12,164 DEGs in A-subgenome and 9954 DEGs in B-subgenome were found to be differentially expressed across combinations. Highest number of DEGs (1605) were mapped on chromosome B03 followed by A03 (1603 DEGs) which indicated that highest number of DEGs were mapped on homologous chromosomes (A03/B03) followed by A06/B06 of both subgenomes (Figure 2a).

A core set of 1093 DEGs showed similar expression patterns in all three genotypes. Some DEGs were also detected which commonly expressed between combination of any two genotypes such as 642 DEGs in GPBD 4 vs. ICGV 13208; 762 DEGs in ICGV 13208 vs. TAG 24; 686 DEGs in GPBD 4 vs. TAG 24. A total of 439 DEGs showed similar expression pattern in GPBD 4 and introgression line ICGV 13208 (Figure 2b).

In GPBD 4 control vs. GPBD 4 stressed samples, total 5056 DEGs (2110 upregulated; 2945 downregulated) were transcriptionally active across stages. The expression trend of up and downregulation was high at 50DPI with 1764 DEGs (724 upregulated; 1040 downregulated). The trend of upregulation was more intense from 1DPI to 35 DPI. A total of 284 DEGs were expressed at 1DPI (253 upregulated and 34 downregulated) (Figure 2c; Figure S1). In ICGV 13208 control vs. ICGV 13208 stressed, a total of 8039 DEGs were expressed (4156 upregulated; 3883 downregulated).

In case of ICGV 13208 control vs. ICGV 13208 stressed samples, the highest number of DEGs (3856) (1756 downregulated; 2103 upregulated) were expressed at 2DPI. The total number of DEGs were decreased with increasing DPI in ICGV 13208 and therefore, only 306 DEGs (196 downregulated; 110 upregulated) were expressed (Figure 2d; Figure S1). In TAG 24 control vs. TAG 24 stressed, a total of 5663 DEGs (2983 upregulated; 2680 downregulated) were expressed. At 1DPI, a total of 1548 DEGs were expressed, of which 608 downregulated and 940 were upregulated, while 1815 DEGs (1056 upregulated; 759 downregulated) were expressed at 2DPI (Figure 2e; Figure S1).

**Figure 3:**
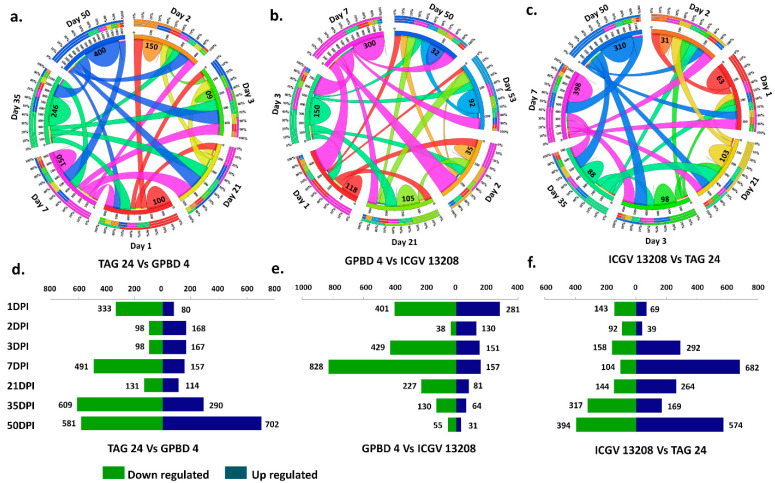
An overview of differentially expressed genes between resistant (GPBD 4, ICGV 13208) and susceptible (TAG 24) genotypes at various stages of disease development (1DPI, 2DPI, 3DPI, 7DPI) and symptom development (21DPI, 35DPI, 50DPI) under *N. personata* infection. Circos plot represents overlapping and specific response of DEGs against *N. personata* at all stages for combinations: (**a**) TAG 24 vs. GPBD 4, (**b**) GPBD 4 vs. ICGV 13208 and (**c**) ICGV 13208 vs. TAG 24. Day wise total up- and downregulated DEGs between resistant and susceptible genotypes shown in (**d**) TAG 24 vs. GPBD 4, (**e**) GPBD 4 vs. ICGV 13208, (**f**) ICGV 13208 vs. TAG 24.

A day-wise overlap between resistant and susceptible samples at all stages was also analyzed. Largest fraction of DEGs were unique at 50DPI for TAG 24 stressed vs. GPBD 4 stressed (400 DEGs) followed by 35DPI (246 DEGs), 7DPI (150 DEGs), 1DPI (100 DEGs), and 2DPI (150 DEGs) (Figure 3a). In TAG 24 stressed vs. ICGV 13208 stressed combination, the largest fraction of DEGs were unique at 7DPI (300 DEGs), followed by 3DPI (150 DEGs), 1DPI (115 DEGs), and 35 DPI (92 DEGs) (Figure 3b). In GPBD 4 stressed vs. ICGV 13208 stressed combination, largest fraction of unique DEGs were expressed at 7DPI (398 DEGs) followed by 50DPI (310 GEGs), and at 21DPI (103 DEGs) (Figure 3c). Overall these results are representing fractions of unique and overlapping DEGs expressed at various stages between resistant and susceptible genotypes.

The expression levels of DEGs between resistant and susceptible genotypes were compared to identify the upregulated and downregulated DEGs upon *N. personata* infection. The highest number of DEGs (4019) across stages were expressed between TAG 24 stressed vs. GPBD 4 stressed (1678 upregulated and 2341 downregulated). The expression trend of upregulation was high at 50 DPI where total 702 DEGs were upregulated and 581 DEGs were downregulated. At 1DPI (333 downregulated), 7DPI (491 downregulated), and 35DPI (609 downregulated) (Figure 3d). In TAG 24 stressed vs. ICGV 13208 stressed, a total of 3007 DEGs were expressed among all samples from all stages (895 upregulated and 2108 downregulated). At 7 DPI, downregulation trend was intense, with 828 DEGs downregulated. Interestingly, the trend of upregulated DEGs was periodic, as the number of upregulated DEGs continuously decreased with increasing number of DPI. For instance, 50DPI the expression trend was very poor with only 31 upregulated and 55 downregulated DEGs (Figure 3e). In GPBD 4 stressed vs. ICGV 13208 stressed combination; at 7DPI and 50DPI, 682 and 574 DEGs were highly upregulated, respectively. However, at 2 DPI only 131 DEGs (92 downregulated and 39 upregulated) were expressed (Figure 3f).

### 2.3. DEGs Expressed in Each Genotype under Control vs. Stressed

Plants respond to pathogen attack by establishing a highly coordinated series of molecular, cellular and tissue-based defense barriers as ample transcript reprogramming occurs in response to a pathogen. We identified important disease resistant genes expressed under stressed condition in resistant and susceptible genotypes when compared with their respective controls. Four different clusters of DEGs identified in three combinations of ICGV 13208 controls vs. ICGV 13208 stressed, GPBD 4 control vs. GPBD 4 stressed and TAG 24 control vs. TAG 24 stressed. In cluster I, large fraction of DEGs was downregulated in susceptible genotype and upregulated in resistant genotypes.

**Figure 4:**
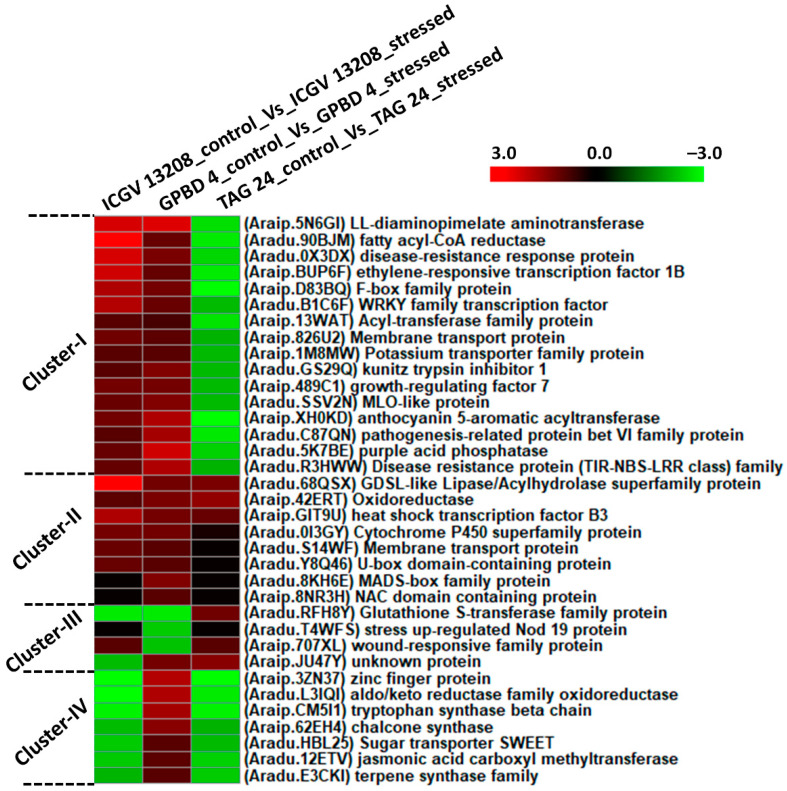
Expression profiles of differentially expressed genes stressed condition between control and stressed samples of each genotype. The clustering of genes was performed using the log2 fold change for each DEG under three combinations (ICGV 13208 control vs. ICGV 13208 stressed, GPBD 4 control vs. GPBD 4 stressed, and TAG 24 control vs. TAG 24 stressed).

DEGs included LL-diaminopimilate aminotransferease (*Araip.5N6GI*), aldo-keto reductase family oxidoreductase (*Aradu.L3IQI*) and fatty acyl-co-A reductase (*Aradu.90BJM*) were downregulated in TAG 24 and upregulated in GPBD 4 and ICGV 13208. Disease resistance response proteins (*Aradu.0X3DX*) and ethylene responsive transcription factor (*Araip.BUP6F*) were downregulated in susceptible genotype and upregulated in resistant genotypes. Important disease resistant proteins (TIR-NBS-LRR) (*Aradu.R3HWW*), MLO-like proteins (*Aradu.SSV2N*), kunitz trypsin inhibitor (*Aradu.GS29Q*), growth regulating factor (*Araip.489C1*), also anthocyanin pigment producing gene anthocyanin 5-aromatic acyltransferase (*Araip.XH0KD*) were downregulated in susceptible genotype. Pathogenesis related proteins (*Aradu.C87QN*), and purple acid phosphatase (*Aradu.5K7BE*) were found upregulated in resistant genotypes. In cluster II, DEGs were highly upregulated in resistant as well as susceptible genotypes. Included MADS-box (*Aradu.8KH6E*) family proteins and NAC domain containing protein (*Araip.8NR3H*) were highly upregulated in resistant as well as susceptible genotypes. In cluster III, DEGs upregulated in susceptible genotypes including stress upregulated Nod 19 protein (*Aradu.T4WFS*), and wound responsive family protein (*Araip.707XL*). In cluster IV, a large fraction of DEGs was downregulated in TAG 24 and ICGV 13208. Included are zinc finger protein (*Araip.3ZN37*), tryptophan synthase (*Araip.CM5I1*), chalcone synthase (*Araip.62EH4*), sugar transporters SWEET genes (*Aradu.HBL25*), and secondary metabolites producing genes such as jasmonic acid carboxyl methyltransferase (*Aradu.12ETV*) and terpene synthase (*Araip.E734B*) (Figure 4; Table S2).

### 2.4. Differentially Expressed Genes between Resistant and Susceptible Genotypes at Disease Development (DD) Stage

**Figure 5:**
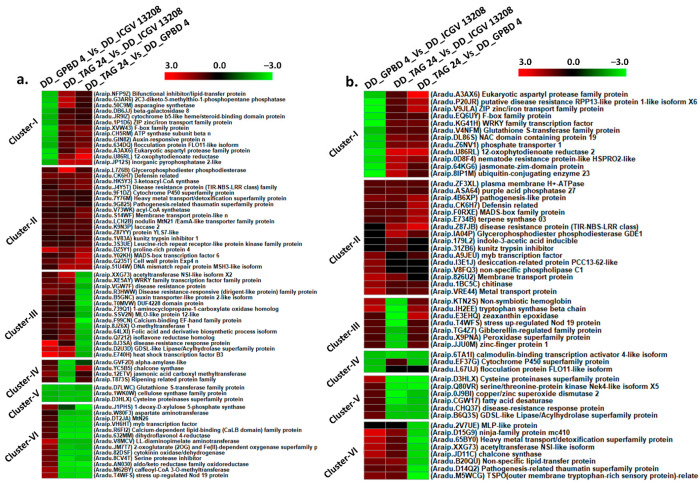
Expression profiles of differentially expressed genes and clustering of DEGs under stressed condition between resistant and susceptible genotypes. The clustering of genes was performed using the log2 fold change for each DEG under three combinations (GPBD 4 vs. ICGV 13208, TAG 24 vs. ICGV 13208, and TAG 24 vs. GPBD 4) under (**a**) disease development (DD) stage and (**b**) symptom development (SD) stage.

Expression trends during disease development (DD) and symptom development (SD) stages identified six different clusters in both stages. In cluster I, 12 DEGs showed downregulation in GPBD 4 vs. ICGV 13208 combination at DD stage, however, were found upregulated in TAG 24 vs. ICGV 13208 and TAG 24 vs. GPBD 4 combinations. The genes of this cluster included putative disease resistance RPP13 (*Aradu.P20JR*), ZIP transport family proteins (*AraipV9JLA*), F-box proteins (*Aradu.EQ6UY*), WRKY TFs (*Aradu.KG41H*), NAC domains (*Araip.DL86S*), nematode resistance proteins (*Araip.0D8F4*), jasmonates-zim-domain (*Araip.64KG6*), ubiquitin-conjugating-enzyme (*Araip.8IP1M*). However, cluster II showed induced expression of DEGs in all combinations, comprising the genes such purple acid phosphatase (*Aradu.ASA64*), pathogenesis related proteins (*Araip.4B6XP*), defensins (*Aradu.CK6H7*), MADS box (*Araip.F0RXE*), terpene synthases (*Araip.E734B*), disease resistance proteins (TIR-NBS-LRR) (*Aradu.Z87JB*), indole 3-acetic acid (*Araip.179L2*), kunitz trypsin inhibitor (*Araip.31ZB6*), MYB transcription factor (*Aradu.A9JEU),* chitinases (*Aradu.1BC5C*), desiccation related proteins (*Aradu.I3E1J*). DEGs in cluster III showed upregulation in GPBD 4 vs. ICGV 13208 and TAG 24 vs. GPBD 4 combinations. The genes in this cluster included zeaxanthin epoxidase (*Aradu.E3EHQ*), stress upregulated Nod 19 (*Aradu.T4WFS*), gibberellin regulated family protein (*Araip.TG4Z7*), peroxidase superfamily protein (*Aradu.X9PNA*), zinc-finger protein (*Araip.JJU0M*). In cluster IV, three DEGs were downregulated in all three combinations. In cluster V, the expression trend showed downregulation in TAG 24 vs. ICGV 13208 and TAG 24 vs. GPBD 4. The genes in this cluster consisted cysteine proteinases (*Araip.D3HLX*), serine threonine protein kinases (*Araip.Q80VR*), copper/zinc superoxide dismutase (*Araip.0J9BI*), disease resistance response protein (*Araip.CHQ37*), The expression trend in cluster VI was upregulated in combinations of GPBD 4 vs. ICGV 13208 and TAG 24 vs. ICGV 13208. The fraction of DEGs in this cluster included MLP-like proteins (*Aradu.2V7UE*), chalcone synthase (*Araip.JD11C*), lipid transfer protein (*Aradu.B20QU*), pathogenesis related protein (*Aradu.D14Q2*) (Table S3; Figure 5a).

### 2.5. Differentially Expressed Genes between Resistant and Susceptible Genotypes at Symptom Development (SD) Stage

The DEGs in SD stage were classified into six clusters on the basis of their expression pattern. In cluster I, fraction of DEGs upregulated in TAG 24 vs. ICGV 13208 and TAG 24 vs. GPBD 4 combinations. The genes in cluster I included lipid transfer proteins (*Araip.NFP9Z*), beta galactosidase (*Aradu.DB6JJ),* cytochrome b5-like heme/steroid binding domain (*Aradu.JR9IZ*), ZIP transport proteins (*Aradu.1P1D6*), ATP synthase beta subunit (*Araip.CH5RM*), auxin responsive protein (*Aradu.B5GNC*), eukaryotic aspartyl protease (*Aradu.A3AX6*), inorganic pyrophosphate (*Araip.JP12S*). Cluster II comprised the DEGs upregulated across three combinations. Cluster II included defensins (*Aradu.CK6H7*), disease resistance protein (*TIR-NBS-LRR*) (*Aradu.J4Y5T*), pathogenesis related (PR) proteins (*Aradu.9G825*), acyl-CoA-synthase (*Aradu.V73WK*), leucine rich receptor (*Aradu.3S3UE*), proline reach protein (*Aradu.DZ5Y1*) and MADS box (*Aradu.Y02KH*). While, in cluster III, the DEGs were upregulated in GPBD 4 vs. ICGV 13208 and TAG 24 vs. GPBD 4 combinations. This cluster consisted of WRKY transcription factor (*Aradu.XE5AY*), disease resistance protein (*Araip.VGW7F, Aradu.R3HWW*), auxin transporters (*Aradu.B5GNC*), MLO-like protein (*Aradu.SSV2N*), calcium binding protein (*Aradu.F99CN*), isoflavone reductase homolog (*Aradu.Q7212*) and heat shock transcription factors (*Aradu.E740H*). However, in cluster IV, four DEGs were upregulated in TAG 24 vs. GPBD 4 and GPBD 4 vs. ICGV 13208. The genes in this cluster included chalcone synthase (*Aradu.YC5B5*), jasmonic acid carboxyl methyltransferase (*Aradu.12ETV*) and ripening related protein family (*Aradu.T873S*). The genes in cluster V were downregulated across combinations. The fraction of DEGs in cluster VI were upregulated in GPBD 4 vs. ICGV 13208 combination. The genes in this cluster included myb transcription factor (*Araip.VH6HT*), serine protease inhibitor (*Aradu.8CV4T*), caffeoyl-CoA-3-O-methytransferase (*Aradu.M62BY*) and stress upregulated Nod 19 protein (*Aradu.T4WFS*) (Table S4; Figure 5b).

### 2.6. Differentially Expressed Genes from Previously Reported QTL Regions for LLS Resistance

The pattern of gene expression was studied for the genes reported in the previously identified QTL regions conferring resistance to LLS mapped on chromosome A02 [22] and A03 [7]. In the present study, we used an integrated approach of genomics and transcriptomics identified differentially expressed genes in these QTL regions for LLS resistance.

In total, 13 DEGs were differentially expressed in a region of 1.4 Mb (0.04 Mb–1.47 Mb) on chromosome A02 [22]. Log_10_ transformed FPKM values were used for visualization of expression pattern of DEGs on heatmap. Of these 13 genes, 5 DEGs, including tetratricopeptide repeat (TPR) superfamily proteins (*Aradu.56PSF; Arahy.UKR13J*), putative disease resistance RPP-like proteins (*Aradu.P20JR; Arahy.A30FPN*), pentatricopeptide repeat (PPR) (*Aradu.VA2KB; Arahy.MTM7TL*), chitinases A (*Aradu.1BC5C*), NADP dependent alkenal double bond reductase (*Aradu.JZB0C*) were upregulated in GPBD 4 and ICGV 13208. The putative disease resistance RPP13-like protein (*Aradu.P20JR*) was consistently upregulated (with log2 fold change > 3) in disease development stage and symptom development stage in resistant genotypes and downregulated in susceptible TAG 24. A fraction of eight DEGs from this QTL region were upregulated in ICGV 13208 and TAG 24. Zinc finger proteins and AN1 domain stress associated proteins (*Aradu.PH3JT*), calmodulin binding heat shock proteins (*Aradu.MS73B*), late embryogenesis abundant protein (*Aradu.KAB2Z*), heavy metal transport/detoxification proteins (*Aradu.S0307*), MLO-like proteins (*Aradu.5Y217*), CAAX prenyl proteases (*Aradu.F2ZNU*), ubiquitin protein ligase (*Aradu.P4SDG*) and calcium binding EF family protein (*Aradu.4GK35*).

**Figure 6:**
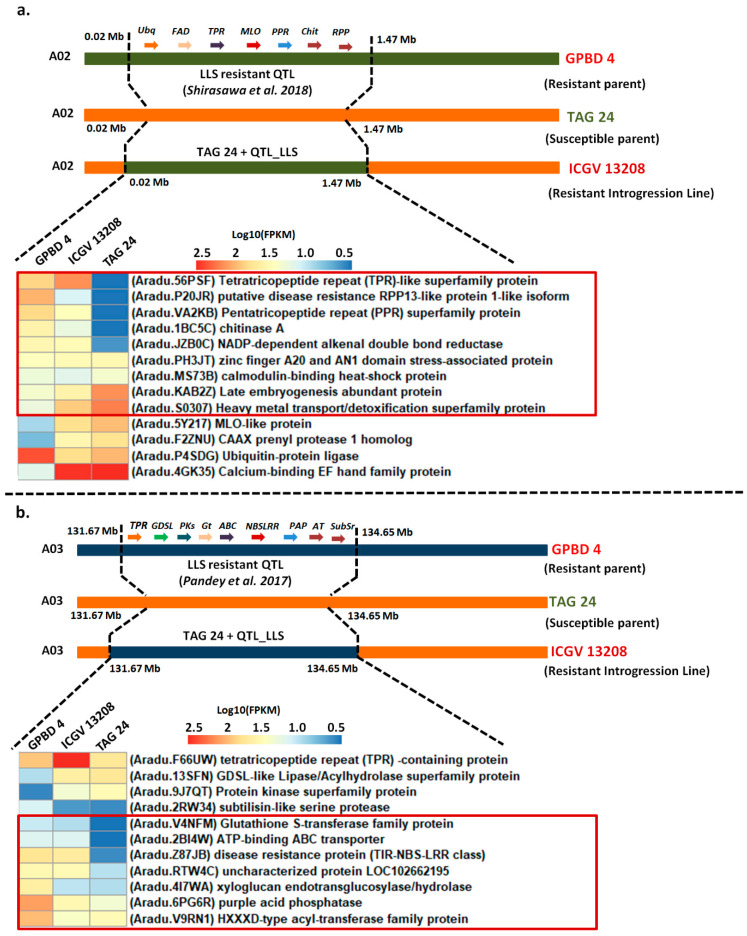
Differentially expressed genes in QTL regions for LLS resistance. (**a**) Expression of DEGs in QTL region on chromosome A02 of 1.4 Mb (0.04–1.47 Mb). (**b**) Expression of DEGs in QTL region on chromosome A03 of 2.98 Mb (131.67 Mb–134.65 Mb). The genes in the red box following a similar expression pattern in introgression line (ICGV 13208) and resistant donor (GPBD 4).

The MLOs are expressed in both disease development and symptom development stage with upregulation trend in TAG 24 and ICGV 13208 and downregulation in GPBD 4. However, late embryogenesis abundant protein was upregulated in all genotypes along with zinc finger stress associated protein (Table S5; Figure 6a).

In the QTL region on chromosome A03, 11 DEGs were transcriptionally active in a genomic region of 2.7 Mb (131.67 Mb–134.65 Mb) identified for LLS resistance [7]. Six DEGs located in this QTL region were downregulated in TAG 24 and upregulated in ICGV 13208 and GPBD 4. The genes included in this genomic region are subtilisin-like serene protease (*Aradu.2RW34*), glutathione S-transferase (*Aradu.V4NFM*), ATP binding ABC transporter (*Aradu.2BI4W*), disease resistance protein (TIR-NBS-LRR) (*Aradu.Z87JB*) and xyloglucan endotransglucosylase (*Aradu.4I7WA*).

The disease resistance protein (TIR-NBS-LRR) (*Aradu.Z87JB; Arahy.R8KUIR*) was consistently upregulated in resistant genotypes and downregulated in susceptible genotypes during disease and symptom development stages. A fraction of DEGs including tetratricopeptide repeat (TPR) protein (*Aradu.F66UW; Arahy.TRXD5D*), purple acid phosphatase (*Aradu.6PG6R; Arahy.II8QNR*) and acyl-transferase family protein (*Aradu.V9RN1; Arahy.K5F7Q0*) were upregulated in GPBD 4 and ICGV 13208 as compared to TAG 24. In both genomic regions (chromosome A02 and A03), the disease resistance proteins were consistently upregulated in GPBD 4 and ICGV 13208 (Table S5; Figure 6b). Further investigation is required to make sure that the phenotypic change is because of the differential gene expression in identified candidate genes and not difference in gene function.

### 2.7. Gene Annotations, GO (Gene Ontology) Term, and Pathway Analysis

Genes exhibiting differential expression patterns were categorized in a various GO category (Figure S2). Gene ontology analysis functionally annotated a total of 1150 DEGs in A-subgenome. The highest number of DEGs were assigned to principle category (GO:0008150) biological process (3561) followed by (GO:0003674) molecular function (3898) and (GO:0005575) cellular component (3333). The highest number of DEGs were assigned to subcategories (GO:0003824) catalytic activity (2887), (GO:0008152) metabolic process (2722), (GO:0005488) binding (2105), (GO:0031224) membrane part (1621). Interestingly, 519 DEGs were annotated to organic cyclic compounds synthesis process (GO:1901360), 507 DEGs assigned to cellular aromatic compound metabolic process (GO:0006725) which are part of signaling mechanisms in response to a pathogen (Table S6). Similarly, GO analysis functionally annotated 1181 DEGs in B-subgenome. The highest 3703 DEGs were assigned to principal categories biological process (GO:0008150) followed by 4077 molecular function (GO:0003674), 3448 cellular components (GO:0005575). In case of sub-categories, the highest number of DEGs were assigned in 2986 catalytic activities (GO:0003824), 2822 in metabolic process (GO:0008152), 2459 DEGs assigned in cellular process (GO:0009987), 2045 organic substance metabolic process (GO:0071704), 2202 DEGs assigned to binding (GO:0005488) and 1707 DEGs to membrane part (GO:0005575) (Figure S2; Table S7).

**Figure 7:**
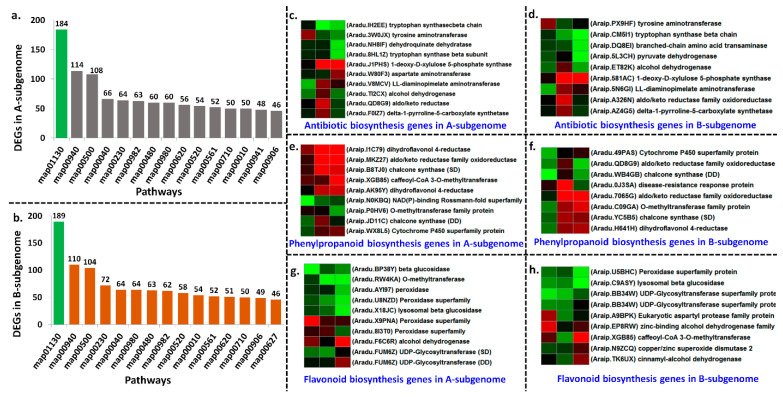
Pathways triggered in response to *N. personata* infection in both subgenomes and expression of major DEGs triggered biochemical pathways in both subgenomes. (**a**) Major pathways triggered in *A*-subgenome, (**b**) major pathways triggered in *B*-subgenome, (**c**) DEGs involved in antibiotic biosynthesis from A-subgenome (**d**) DEGs involved in antibiotic biosynthesis from B-subgenome, (**e**) DEGs involved in phenylpropanoid biosynthesis from A-subgenome, (**f**) DEGs involved in phenylpropanoid biosynthesis from B-subgenome, (**g**) DEGs involved in flavonoid biosynthesis from A-subgenome, (**h**) DEGs involved in flavonoid biosynthesis from B-subgenome. Some genes were expressed in both disease development (DD) and symptom development (SD) stages.

A total of 136 unique pathways were triggered in A-subgenome including biosynthesis of antibiotics, phenylpropanoid biosynthesis, flavonoid biosynthesis, terpenoid backbone biosynthesis and streptomycin biosynthesis (Tables S8 and S9; Figure 7a). A total of 135 pathways were triggered in B-subgenome including biosynthesis of antibiotics, phenylpropanoid biosynthesis, flavonoid biosynthesis, tryptophan metabolism (Tables S10 and S11; Figure 7b). In both subgenomes, common pathways were triggered with common set of genes such as secondary metabolite biosynthesis, antibiotic biosynthesis, streptomycin and tetracycline, cutin and suberine wax biosynthesis, starch sucrose metabolism, pyruvate metabolism, and T cell receptor signaling pathways. Interestingly, 10 DEGs impacted >20 pathways and 18 DEGs impacted >10 pathways in A-subgenome (Table S12). Similarly, 8 DEGs impacted >20 pathways and 20 DEGs impacted >10 pathways in B-subgenome (Table S13).

### 2.8. Joint Pathways Triggered by Homologous Chromosomes in Both Subgenomes upon LLS Infection

Substantial homologous transcript reprogramming in both subgenomes under *N. personata* infection revealed similar genomic footprints in A- and B-subgenomes for LLS resistance. We observed similar set of genes triggered similar pathways in both subgenomes under *N. personata* infection. The major pathways such as biosynthesis of antibiotics, phenylpropanoid biosynthesis, and flavonoid biosynthesis were triggered by similar set of DEGs in both subgenomes. In case of antibiotic biosynthesis pathway, the DEGs such as tryptophan synthase, tyrosine amino transferase, LL-diaminopimilate aminotransferase, delta-1-pyroline 5-carboxilate, alcohol dehydrogenase, 1-deoxy-D-xylulose5-phosphate synthase were expressed in both subgenomes to trigger the antibiotic biosynthesis pathway (Table S14; Figure 7c,d). In case of phenylpropanoid biosynthesis, UDP-glycosyltransferase, peroxidases, lysosomal beta glucosidase DEGs were upregulated in both subgenomes (Table S15; Figure 7e,f). Similarly, in case of flavonoid biosynthesis pathway, cytochrome P450 protein, chalcone synthase, dihydroflavonol 4-reductase, O-methyltransferase protein were upregulated in both subgenomes in resistant genotypes to trigger flavonoid biosynthesis (Table S16; Figure 7g,h). Overall, these results indicated that both subgenomes in tetraploid groundnut showed response to *N. personata* infection.

### 2.9. Validation of Differentially Expressed Genes Using qRT-PCR

Validation of the differentially expressed genes was carried out using quantitative real time polymerase chain reaction (qRT-PCR).

**Figure 8:**
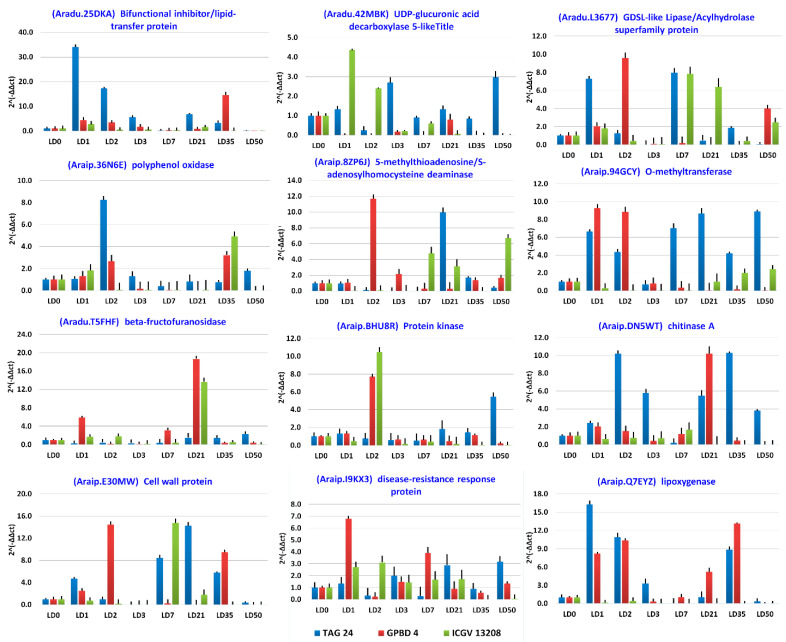
Validation of differentially expressed genes using qRT-PCR between resistant and susceptible genotypes at disease development (1DPI, 2DPI, 3DPI, 7DPI) and symptom development (21DPI, 35DPI, 50DPI) stages.

Gene expression profile of DEGs with log2 fold change >3.0 and <−3.0 for respectively upregulated and downregulated DEGs was generated upon *N. personata* infection in all 48 samples of TAG 24, ICGV 13208 and GPBD 4 genotypes at disease development and symptom development stages. The information on primer sequences of forward and reverse primers of each gene is provided in Table S17. The values recorded at 0DPI were considered as control to study the comparative induced or repressed expression of DEGs. Among 12 genes validated, *Aradu.L3677, Aradu.T5FHF* and *Araip.E30MW* showed induced expression under stress at symptom development stage in resistant genotypes. Among these genes *Aradu.L3677* encoding for GDSL-like lipase/acylhydrolase was shown upregulation in resistant genotypes GPBD 4 and ICGV 13208 at 50DPI when compared with the susceptible it showed upregulation in TAG 24 at 7DPI when compared with control. Similarly, *Aradu.T5FHF* encoding beta-fructofurosidase showed highly induced expression in GPBD 4 and ICGV 13208 at 21DPI under *N. personata* infection. Highly induced expression was observed for *Araip.E30MW* encoding for cell wall protein at 35DPI in GPBD 4 however downregulation in TAG 24 and ICGV 13208 at all stages. The gene *Araip.I9KX3* encoding for disease resistance response protein was differentially expressed in all genotypes at all stages. During disease development stages 1DPI and 7DPI, it was highly (7.1- and 5.2-folds, respectively) upregulated in GPBD 4. However, at 2DPI it was upregulated in ICGV 13208 (3.7-fold) and 50 DPI it was upregulated (3-fold) in TAG 24. Chitinases (*Araip.DN5WT*) showed upregulation in ICGV 13208 at 2DPI (~90-fold) when compared with control. Protein kinase superfamily proteins (*Araip.BHU8R*) showed induced expression at 2DPI in GPBD 4 and ICGV 13208 (9.2- and 10.4-folds, respectively) and at 50DPI in TAG 24 (5.7-fold). O-methyltransferase (*Araip.94GCY*) showed induced expression at 21DPI, 35DPI and 5DPI (40.0-, 30.0- and 20.0-folds, respectively) when compared with control. 5-methylthioadenosine/S-adenosylhomocysteine deaminase (*Araip.8ZP6J*) sowed induced expression at 2DPI (12.3-fold) in GPBD 4, at 21DPI (10.4-fold) in TAG 24 and at 50DPI (6.5-fold) in ICGV 13208. Polyphenol oxidase (*Araip.36N6E*) showed induced expression (22.3-fold) at 2DPI in susceptible TAG 24 and resistant GPBD 4 (7.6-fold). UDP-glucuronic acid decarboxylase (*Aradu.42MBK*) was upregulated at 1DPI and 2 DPI (4.5- and 2.5-folds, respectively) in GPBD 4 and induced expression at 3DPI and 50DPI (2.9- and 3.0-folds) in TAG 24. Lipid-transfer protein/seed storage protein (*Aradu.25DKA*) showed induced expression at 1DPI and 2DPI (32.5- and 19.2-folds) in TAG 24, at 35DPI (15.2-fold) in GPBD 4. The results of qRT-PCR showed similar expression patterns with high-throughput RNA-seq data upon LLS infection (Figure 8).

## 3. Discussion

Foliar diseases such as early leaf spot (ELS), late leaf spot (LLS) and rust often occur together leading to 50–70% yield loss in groundnut. The circular dark spots of the fungus *N. personata* spreads on leaves and it spreads on stem and pegs and affects seed and haulm quality under heavy infection [19,31]. During last decade, several genetic mapping studies were conducted to discover QTLs linked to late leaf spot resistance and the major effect QTLs were discovered on A02 and A03 chromosomes [4,7,19,20,32]. The linked markers were also deployed in MABC to develop resistant varieties using GPBD 4 as donor parent [6,9]. High-quality reference genomes of diploid [33,34] and tetraploid [2,3,4] are important genomic resources for groundnut genomics and breeding. In the present study, the comparative transcriptome analysis was performed between a resistant donor GPBD 4, susceptible a recurrent parent TAG 24 and a MABC derived resistant introgression line ICGV 13208 to identify differentially expressed genes in introgression regions and across the genome.

The objective of this study was to identify the DEGs from the QTL region which was transferred using marker-assisted backcrossing in introgression line (ICGV 13208) using the LLS resistance donor (GPBD 4) and the differential expression pattern of the genes among resistant and susceptible genotypes under *N. personata* infection. The transcriptome analysis unraveled the substantial transcriptome changes in resistant genotypes, GPBD 4 and ICGV 13208, and susceptible genotypes TAG 24 under *N. personata* infection at seven stages (1DPI, 2DPI, 3DPI, 7DPI, 21DPI, 35DPI and 50DPI) in 48 samples. Of the seven stages, four stages belong to disease development (1DPI, 2 DPI, 3DPI and 7 DPI) and three stages (at 21DPI, 35DPI and 50DPI) as symptom development. Recently, an attempt was made for transcriptome analysis discovered DEGs for rust caused by *Cercospora arachidicola* [28] and early leaf spot (ELS) caused by *Puccinia arachidis* [29] while no such study for LLS resistance. In the present study for LLS resistance, a total of 1484 million RNA sequencing reads were generated for 48 samples and mapped on A- and B-subgenome with average mapping percentage 77.5% and 79.8%, respectively. Therefore, around 92% percent of total filtered reads were mapped on both subgenomes. The ELS study generated 91.7 million reads RNA-seq data for resistant GPBD 4 and susceptible JL 24 at 24 h post inoculation of fungus *C. arachidicola* while rust study generated 86.3 million reads for resistant GPBD 4 and susceptible JL 24 at 24 h post inoculation of *P. arachidis*. We have selected more stages and genotypes and generated more data for LLS transcriptome analysis than the previous transcriptome studies for foliar fungal diseases.

We targeted the discovery of differentially expressed candidate resistance genes from reported QTL genomic regions on chromosomes, A02 and A03 for LLS resistance. In QTL region on chromosome A02, the expression of putative disease resistance RPP13-like protein (*Aradu.P20JR*) showed upregulation (34 folds) in GPBD 4 as compared to TAG 24. However, the expression of *Aradu.P20JR* showed downregulation in ICGV 13208 (by 34 folds) when compared with resistant GPBD 4. In QTL region on chromosome A03, the expression of disease resistance protein (TIR-NBS-LRR) (*Aradu.Z87JB*) (133776795-133780539) was increased periodically with increasing days post inoculation. Where, the *Aradu.Z87JB* showed upregulation (25.5-fold) more during disease development stage and more (56.7-fold) during symptom development stage in GPBD 4 and ICGV 13208 as compared with TAG 24. It is important to note that the disease resistant NBS-LRR genes were also reported upregulated in ELS resistant GPBD 4 (4.3-fold) when compared with susceptible JL 24 for ELS disease under *C. arachidicola* infection [28] in groundnut. Similarly, under infection of rust causing *P. arachidis* infection the NBR-LRR class showed upregulation (3.3-fold) in rust resistant GPBD 4 when compare with susceptible JL 24 [28] groundnut. Therefore, the disease resistance NBS-LRR class from QTL regions can be used for improving the late leaf spot resistance in important groundnut cultivars in addition to two other foliar fungal diseases, rust and ELS resistance.

Leaf spot diseases are most severe in the fields where groundnut is grown in the same field in consecutive years, in rainy weather and high humidity [35]. Yield loss occurs due to defoliation of diseased leaflets under heavy *N. personata* invasion. Defoliation reduces healthy leaf area and affects the rate of photosynthesis and weakens the stems and pegs causing pods to fall off during up-rooting. If the leaf spot is not controlled in initial stage of disease development, defoliation level exceeds 50 percent and yield loss also exceeds 50 percent or more [36]. In the present study, we found genes responsible for senescence showing downregulation in susceptible genotypes (TAG 24) as compared to resistant genotype (GPBD 4) (Table S18; Figure S3). The expression trends of ethylene responsive factor (ERF) and ethylene responsive nuclear protein (ERF2) showed downregulation in susceptible genotypes during disease development stage (at 1DPI, 3DPI and 7DPI). However, the expression trend of these genes showed upregulation in susceptible genotypes at symptom development stage (21DPI, 35DPI and 50DPI). Moreover, early responsive dehydration (ERD) family proteins, late embryogenesis abundant (LEA) proteins and stress upregulated Nod19 were also found upregulated in susceptible genotypes at symptom development stage. Ethylene involves in activation of senescence associated genes which cause senescence [37]. Prior studies have reported a group of leaf senescence-associated genes (SAGs) [38]. Foliar application of ethylene stimulates leaf senescence, but ethylene biosynthesis inhibitors delay leaf senescence [39]. Downregulation of an ethylene biosynthesis gene in tomato caused decrease in ethylene production and substantially delayed leaf senescence, evidently signifying that ethylene accelerates leaf senescence [40]. In the present study, the ERFs expressed after complete symptom development of late leaf spot, at 50DPI the ERF was highly upregulated in TAG 24 which may have triggered ethylene insensitive (EIN), NACs, abscisic acid (ABA) which results in onset senescence. In addition, ethylene masks the expression of GOLDEN-LIKE2 (GLKs) which stops chloroplast biosynthesis and leaves starts yellowing due to lack of chlorophyll. Ethylene also masks the expression of auxin IAA (indol acetic acid) biosynthesis genes which results in senescence [37].

**Figure 9:**
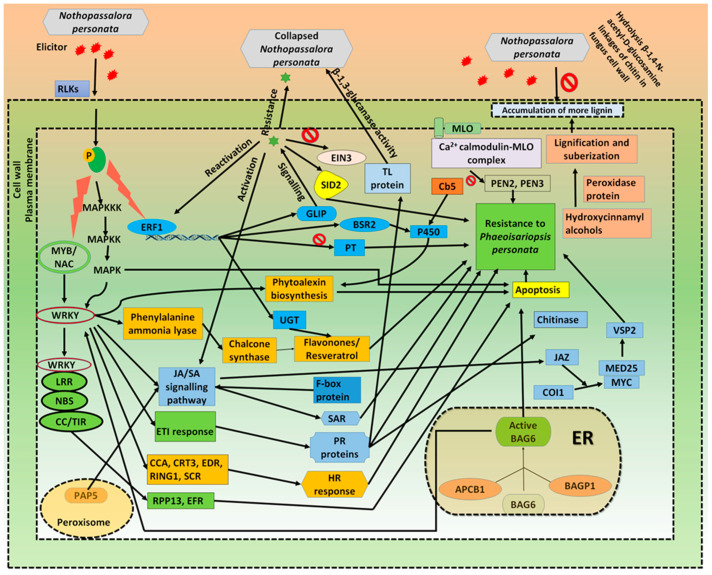
A proposed framework showing host–pathogen cross talks between late leaf spot pathogen (*N. personata*) and groundnut. The illustration represents the various transcription factors/genes/proteins involved in the defense response in groundnut under *N. personata* infection. The response of groundnut to the LLS infection included the substantial transcriptome reprogramming activation of the different interlinked pathways start off by elicitor recognition to the hypersensitive response, resulted in resistance to *N. personata*. The biological pathways include MAPK cascade, transcription factors activation, JA/SA signaling, GLIP signaling and calcium cation signaling. Such cascades triggered the expression of genes involved in the defense response such as regulation of lignification, suberization, R-protein, F-box protein, Pathogenesis-related protein, phytoalexins, flavanones, resveratrol, chitinase and chalcone synthase. These compounds have deteriorating impact on the fungus reproduction and make plant less prone to further infection. During entire defense response, multiple cell organelles are involved such as peroxisomes for phosphatase synthesis, nucleus and endoplasmic reticulum for antifungal protein synthesis.

This transcriptome analysis also provided insights on the genome-wide molecular cross-talks between *N. personata* and *Arachis hypogaea*. Under LLS infection, the receptor like kinase (RLKs) have significant role in plant recognition and infection of *N. personata*. Mitogen activated protein kinase (MAPK) is the signaling cascade widely triggered in response to pathogen infection [41]. For transmitting the response signal by means of phosphorylation, MAPKKK activates MAPKK, and then MAPK [42]. MAPK cascades have critical role in multiple signaling defense responses, including the monitoring of plant defense gene activation through upregulation of WRKY and hypersensitive response (HR) cell death known as apoptosis. Upregulation of WRKY regulates resistance to *N. personata*, needs JA-mediated signal transduction and SA-dependent pathways and thus monitor crosstalk between JA- and SA-regulated disease response pathways [43]. Overexpression of MYB TFs and NAC stimulates the expression of plant PR genes and is regulated by phytohormones, mainly JA and SA followed by triggering systemic acquired resistance (SAR). Upregulation of AtMYB44 leads to resistance against *Pseudomonas syringe* through SA signaling in *Arabidopsis* [44]. The NBS-LRR proteins were upregulated to recognize effectors and trigger the effector trigger immunity (ETI) response together with the interaction of WRKY which results in HR and apoptosis. Similar interactions of a coiled coil (CC)-NB-LRR protein with *HvWRKY1*, imparting resistance to powdery mildew in *Hordeum vulgare* [45]. The infection of *N. personata* activates the ethylene signaling pathway in which ERF1 encodes a transcription factor of the ethylene-responsive element-binding protein (EREBP) family. ERF1 upregulated the GDSL-like lipase (GLIP) and released into the cell wall. The signaling cascade of GLIP occurs through phloem to systemic tissues and causes the reactivation of ERF1 and the breakdown of EIN3, rising SID2 and SA levels in systemic tissues leads to the launching of systemic acquired resistance (SAR) [46,47]. Jasmonic acid is broadly distributed as a natural plant growth regulator and signaling molecule in the plant kingdom. The cross-talks between JA and other plant hormone signaling have vital function in managing plant stress responses [48]. The upregulation of JAZ by action of JA signaling resulted in binding of COI1 and MYC2 to activate the expression of VSP2 mediated by MED25, thus increasing the resistance to plant against wound. Apart from involvement in the hormone metabolism, the cytochrome P450s encoded by Broad-spectrum resistance2 (BSR2) gene was upregulated in plant defense mechanism through their pivotal role in phytoalexin biosynthesis which leads to apoptosis of infected tissues [49]. The glycosylation of the acceptor molecules such as flavanols, flavonoids, saponins, sterols terpenoids, plant hormones is upregulated by UDP-glycosyltransferase protein (UGT) and neutralize xenobiotics, and thus play a crucial role in plant-pathogen interactions [50]. The upregulation of F-box protein encoding genes regulates SA signaling cascade. In transgenic tobacco, overexpression of OsDRF1 (F-box protein encoding gene) caused an increase in disease resistance against *Pseudomonas syringae* pv. tabaci and empowered the expression of defense related genes after salicylic acid treatment [51]. The MLO protein, which is present in the plasma membrane, mediates a Ca^2+^ dependent interaction with calmodulin. The PEN2 and PEN3 act separately in different pathways contributing resistance against pathogen penetration [52,53]. In present study, the MLO protein found as negative regulator of PEN2 and PEN3 pathways contributing to LLS resistance. Upregulation of peroxidase mediates the oxidation of hydroxycinnamyl alcohols into free radical intermediates, phenol oxidation, polysaccharide cross-linking, lignification and suberization. The building up of lignin and phenolic compounds are important physical barriers to impart the resistance in a number of host–pathogen interactions [54]. PAP5, which is localized in the peroxisome, is positively induced during the earlier stages as a component of ROI generation accompanied by JA/SA signaling pathways [55]. The Bcl-2-associated athanogene (BAG6) is vital for basal immunity against the fungal attack by autophagy that coincides with disease resistance. The upregulated eukaryotic aspartyl protease (APCB) processed the inactive BAG6. Ref. [56] demonstrated the autophagy activated by the degradation of BAG6 confers resistance to the necrotrophic fungal pathogen *Botrytis cinerea*. The downregulation of pathogenesis-related thaumatin superfamily protein (TLP) could not act on invading fungi by means of hydrolysis of β-1, 3-glucans in susceptible cultivar [57]. The PR protein such as chitinase involves hydrolysis of β-1, 4-N-acetyl-D-glucosamine linkages of chitin result in the rupture of fungus cell wall. Thus, imparting resistance against *N. personata* infection in the groundnut. Ref. [58] identified the stem rot resistant QTL region harboring genes encoding chitinase enzyme which contribute to fungus cell wall degradation. In addition to these, other secondary metabolites such as phytoalexins, resveratrol synthase, terpene synthase, PR proteins, CCA, CRT3, Phosphate transporter, EDR, RING1, SCR, chalcone synthase, PAL and RPP13-like protein were expressed during defense processes. Overall findings showed that LLS disease has triggered various genes and pathways in groundnut via substantial transcriptome reprogramming against *N. personata* infection. These datasets would be useful genomic resources in understanding the late leaf spot resistance mechanism in groundnut (Figure 9).

In summary, the comparative transcriptome analysis in groundnut identified important differentially expressed genes at disease development and symptom development stages including defoliation. Important disease resistance genes such as RPP13-like protein and NBS-LRR genes in previously reported QTL regions were identified for LLS resistance. The tetraploid gene IDs for a few important DEGs are provided in result section. However, the tetraploid gene IDs for the rest of the genes can be retrieved from peanutbase. Pathway analysis identified important pathways such as antibiotic biosynthesis, flavonoid biosynthesis, phenylpropanoid biosynthesis which were triggered in both subgenomes under *N. personata* infection. Ethylene responsive factors were identified which are highly expressed in susceptible genotypes at symptom development causing defoliation in susceptible genotypes at maturity.

## 4. Materials and Methods

### 4.1. Plant Material and Experimental Conditions

Three groundnut genotypes were used in this study, namely TAG 24, GPBD 4 and ICGV 13208. TAG 24 is an elite groundnut cultivar that is highly susceptible to LLS. GPBD 4 is an elite cultivar that is a well-adapted LLS and rust resistant variety in India, used as a donor parent during marker-assisted backcrossing. ICGV 13208 is the BC_2_F_6_ LLS resistant introgression line with genomic regions imparting resistance to LLS introgressed from donor parent GPBD 4 in the background of recurring parent TAG 24 [27]. In total 15 pots for each genotype were grown in two sets in two separate greenhouses. The seeds were sown in pots (12 cm diameter) filled with 1:1 sterile soil and sand mix. One set was treated as control, i.e., without any inoculation and other was inoculated with spores of *N. personata* at 40 days after sowing (DAS).

### 4.2. Inoculation with Spores of N. personata and Sample Collection

The spores of *N. personata* were collected from the highly susceptible groundnut cultivar TMV 2 in rainy season of year 2016. The brown spot with mass of spores with velvety appearance were usually found on the underside of the leaf. These spores were collected by gentle brushing and the concentrations of the spore suspensions were optimized to 20,000 spores mL^−1^ using a hemocytometer by adding sterile distilled water with few drops of Tween-80 (polyoxyethylene sorbitan mono-oleate) for proper adhesion. Inoculation was done with an atomizer sprayer on the leaves of each plant at 40 DAS in one set and the control plants were not inoculated. For proper disease development, the plants were covered with polythene sheets and were sprayed with distilled water once in every two hours so that humidity of >95% is maintained. The conducive conditions for disease development were maintained for seven days by monitoring the relative humidity and temperature (25–30 °C).

**Figure 10:**
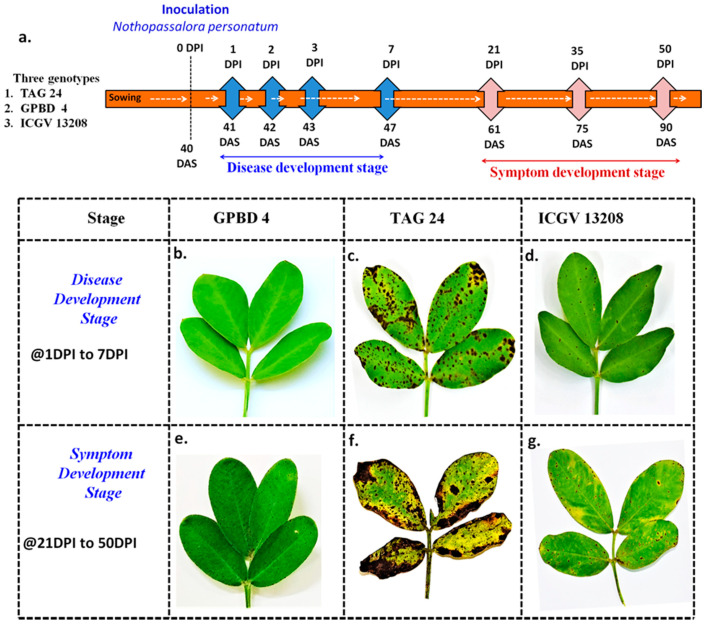
Timeline for sample collection at different days post inoculation (DPI) for LLS transcriptome analysis. (**a**) Illustrates sample collection at seven different stages (1DPI, 2DPI, 3DPI, 7DPI, 21 DPI, 35DPI and 50DPI) from TAG 24, ICGV 13208 and GPBD 4 genotypes, (**b**–**d**) phenotypes of GPBD 4, TAG 24 and ICGV 13208, respectively at symptom development stages. (**e**–**g**) Phenotypes of GPBD 4, TAG 24 and ICGV 13208, respectively at disease development stages.

The leaf tissues were harvested for RNA isolation both from control and inoculated treatments and flash frozen in liquid nitrogen and stored at −80 °C. The samples were collected just before inoculation on 40 DAS, then one day post inoculation (1 DPI) (41 DAS), 2DPI (42 DAS), 3 DPI (43 DAS) and 7 DPI (47 DAS) for studying the disease development in all three genotypes GPBD 4, TAG 24 and ICGV 13208. In order to study the symptom development stages, the samples were collected on 21 days post inoculation (DPI) (61 DAS), 35 DPI (75 DAS) and 50 DPI (95 DAS). In total, 48 samples (8 stages × three genotypes × 2 treatments) were used for RNA isolation and sequencing (Figure 10).

### 4.3. RNA Isolation and Sequencing

Total RNA was isolated from the groundnut leaves using “NucleoSpin^®^ RNA Plant” kit (Macherey-Nagel, Germany) following user’s manual. RNA quality and quantity was determined using Nanodrop 1000 spectrophotometer (Thermo Fisher Scientific Inc, Wilmington, DE, USA) and Bioanalyzer RNA Nano chip (Agilent Technologies, Santa Clara, CA, USA). The RNA samples with 260/280 ratio of 1.8 to 2.1, 260/230 ratio of 2.0 to 2.3 and RNA integrity number (RIN) more than 7.0, were used for mRNA sequencing. The cDNA library was prepared using mRNA-Seq Sample Prep kit (Illumina Inc., San Diego, CA, USA) following manufacturer’s instructions. Poly (A)-containing mRNA was isolated using magnetic beads with oligo (dT) and fragmented into short pieces. These short fragments were used as templates to synthesize first-strand cDNA using reverse transcriptase and random hexamer-primers. The second-strand cDNA was then synthesized using DNA polymerase, dNTPs and RNase H. After completing purification and end repair process, the cDNA fragments were ligated to sequencing adapters. The fragments were then purified and amplified by PCR to obtain the final library followed by purification. Paired-end sequencing was carried out on Illumina HiSeq 2500 platform and raw reads of 100nt were generated. Filtered reads were obtained after running the quality control (QC) using NGS-QC box [59].

### 4.4. Read Alignment, Transcript Abundance and Gene Expression Analysis

Genome assemblies of both the progenitor subgenomes A-subgenome (*A. duranensis*) and B-subgenome (*A. ipaensis*) of cultivated groundnut (*A. hypogaea*) [34] was used as the reference genome for mapping high quality reads and further downstream analysis. The reads were mapped using TopHat2 [60]. Read counts were normalized by calculating the fragments per kilobase of exon per million fragments mapped (FPKM) value for each transcript. Reads were assembled into transfrags using cufflinks v2.1.1 [61]. Transcripts with FPKM ≥ 1 having the maximum number of isoforms were identified in each sample to estimate the transcript abundance in each tissue. DEGs were identified using Cuffdiff [62]. Genes with log2 fold change values of ≥+2 and ≤−2 (up- and downregulated) and False Discovery Rate (FDR) adjusted *p*-value ≤ 0.05 after Benjamini–Hochberg correction for multiple-testing [63] with significance level ‘yes’ were considered as DEGs.

### 4.5. Clustering and Principle Component Analysis (PCA)

Transcripts with abundance > 1 FPKM were used for calculation of pairwise correlation between each sample pair. The function ‘corrl’ implemented in Microsoft office excel 2013 was used to calculate pairwise correlation matrix between 48 samples including 24 control and 24 under *N. personata* infection load at different time points. The pairwise correlation matrix was further used for cluster analysis using R package ‘pheatmap’ version 1.0.12 [61]. Further samples were clustered on the basis of correlation (r) values (ranging 0.0 to 1.0) for each pair of samples. Identified DEGs with log2 fold change ≥ 2 were considered as induced, or ≤ −2 considered as repressed. Log2 transformed FPKM values of the DEGs were further subjected to K-means clustering using Pearson correlation in R package “pheatmap” version 1.0.12 [64]. Different clusters were separated in disease development stage and symptom development stage using ‘cutree’ function implemented in pheatmap.

### 4.6. Quantitative PCR (qPCR) Analysis

To validate the expression analysis of key candidate genes, primers were designed using Primer 3 plus tool (http://www.bioinformatics.nl/cgi-bin/primer3plus/primer3plus.cgi). The alcohol dehydrogenase (*Adh*) gene was used as an internal reference as *Adh* shows highly stable expression across all groundnut tissues as compare to other housekeeping genes such as *14-3-3* [65]. The cDNA was prepared using superscript first strand synthesis followed by second strand synthesis according to the instructions of manufactures (Invitrogen, Carlsbad, CA, USA). The primer efficiency was checked using 10-fold dilution of template (cDNA) on all the primers and the primers with efficiency ranging from 90 to 110% were used further for qPCR or real time PCR (RT-PCR). The qPCR was performed on the Applied Biosystems 7500 Real Time PCR systems using SYBR Green chemistry following the manufacturer’s instructions (Invitrogen, Carlsbad, CA, USA). At least two independent biological replicates and three technical replicates were used for RT-PCR analysis. Data analysis was carried out using the delta Ct method [66].

## Data Availability

The sequencing data have been deposited at National Center for Biotechnology Information Sequence Read Archive (NCBI-SRA) database with the Bio Project ID-PRJNA660596.

## Supplementary Materials

The following are available online at https://www.mdpi.com/article/10.3390/ijms22094491/s1.

## Abbreviations

RLKs: Receptor like kinases, PAP5: Purple acid phosphatases, MYB: MYB transcription factor, NAC: NAC domain protein, GLIP: GDSL like lipase, P450: Cytochrome P450 family protein, Cb5: Cytochrome B5, F-box: F box protein, UGT: UDP glycosyltransferase protein, JAZ: Jasmonate ZIM domain, APCB1: Eukaryotic aspartyl protease family protein, TL: Pathogenesis-related thaumatin superfamily protein, CCA: Circadian clock associated, SCR: Cysteine-rich secretory proteins, PT: Phosphate transporter, PAL: Phenyl alanine ammonia lyase.
