# Supplementary material for: Comparative Transcriptome Analysis Identified Candidate Genes for Late Leaf Spot Resistance and Cause of Defoliation in Groundnut
Source: Int J Mol Sci. 2021 Apr 26;22(9):4491. doi: 10.3390/ijms22094491 (PMC8123497; doi:10.3390/ijms22094491)
Supplement: Supplementary file 1 [file ijms-22-04491-s001.zip › Supplementary Material/ijms-1159347 sup.pdf]

GPBD4

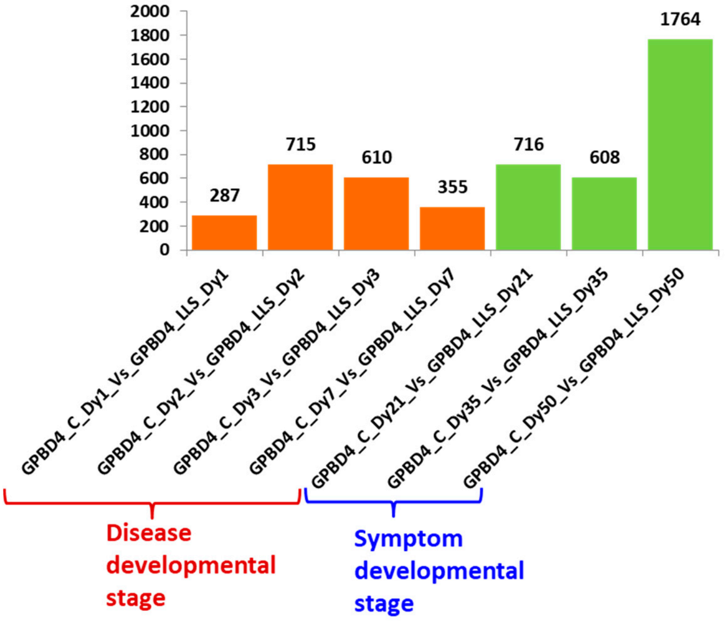

ICGV 13208

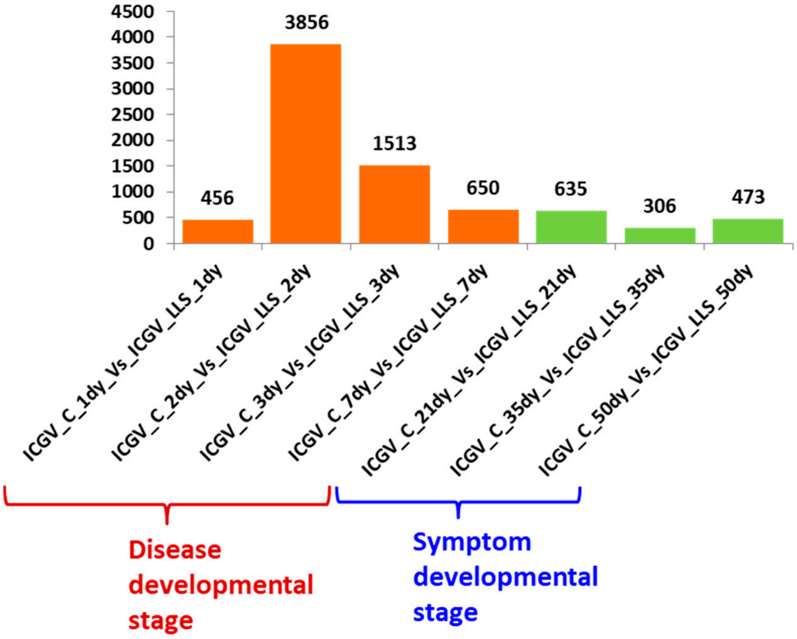

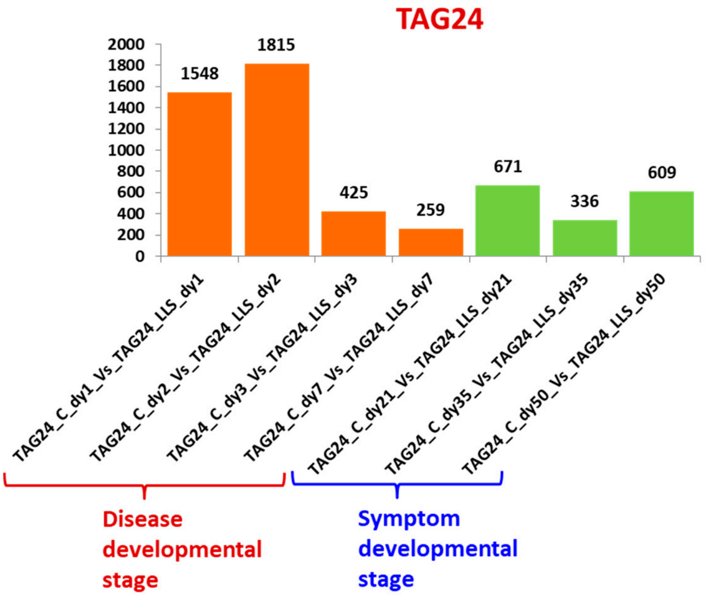

Figure S1. Total number of DEGs (upregulated and downregulated) expressed in resistant (GPBD4 and ICGV13208) and susceptible genotypes (TAG24) in control vs stress condition at 1DPI, 2DPI, 3DPI, 7DPI, 21DPI, 35DPI and 50DPI under P. personata infection.

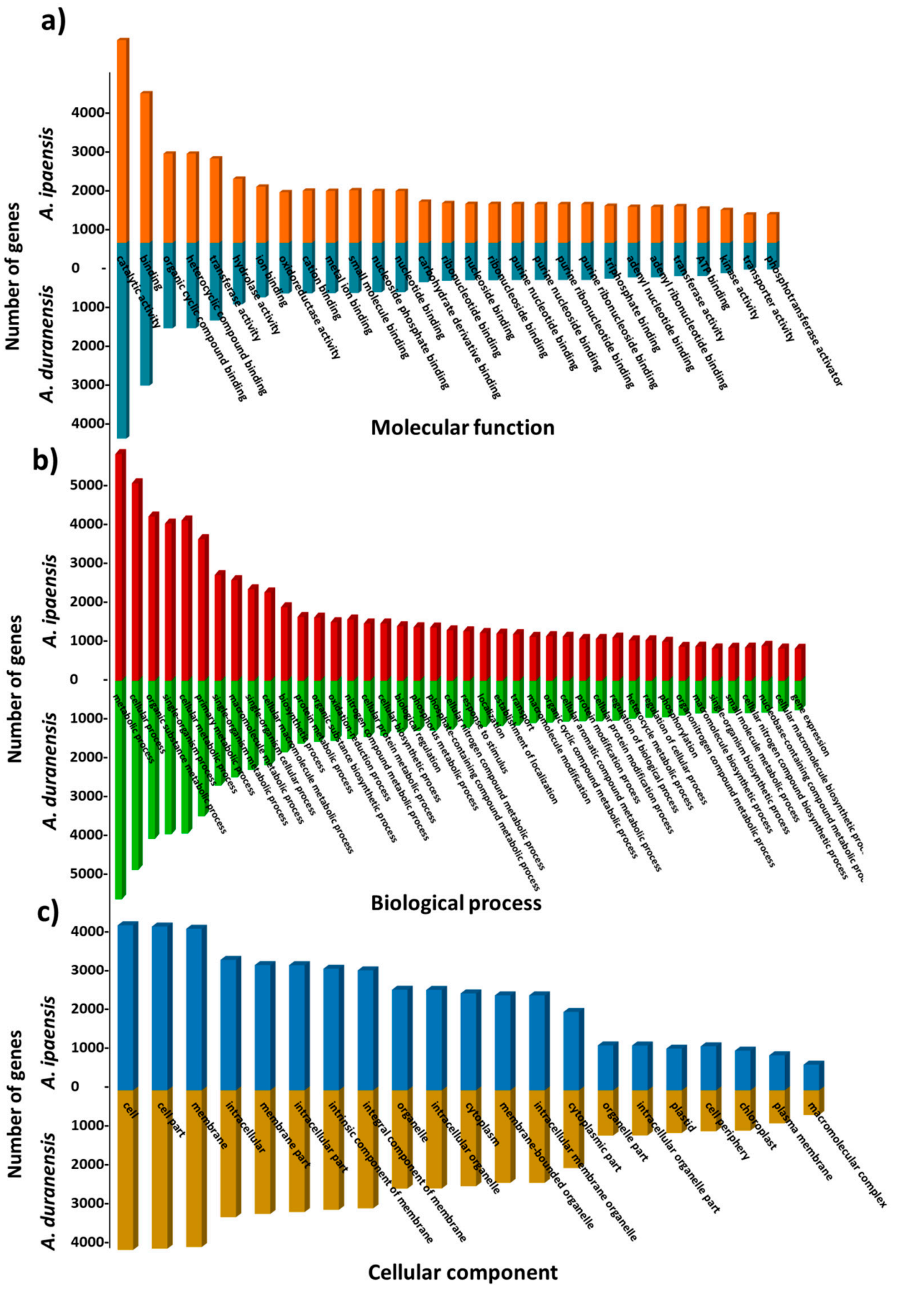

Figure S2. Gene ontology annotation of differentially expressed genes in both subgenomes A. duranensis and A. ipaensis. Bi-directional bar plots representing GO annotations of three major categories and subcategories (**a**) biological process, (**b**) molecular function, (**c**) cellular components.

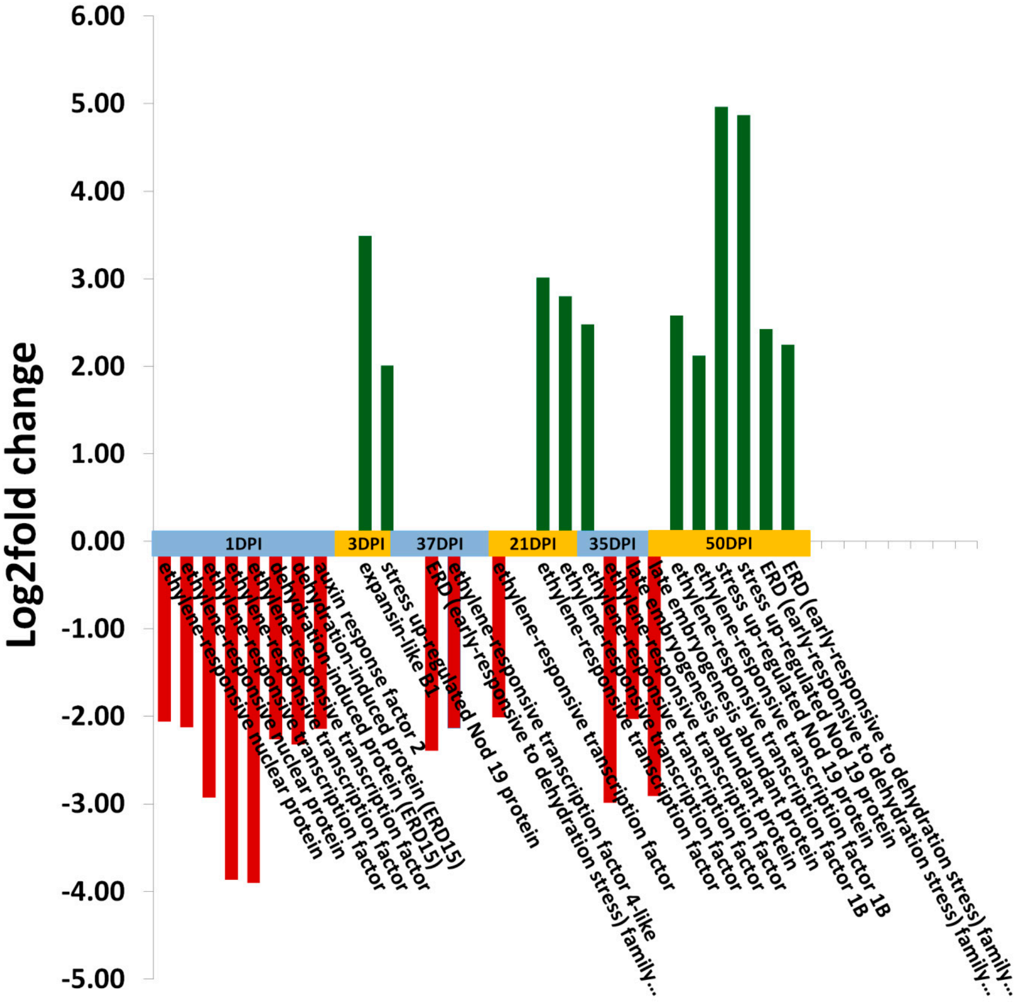

Figure S3. Expression of DEGs involved in ethylene biosynthesis in resistant and susceptible genotypes.
